# Myosin-18B Promotes Mechanosensitive CaMKK2-AMPK-VASP Regulation of Contractile Actin Stress Fibers

**DOI:** 10.1016/j.isci.2020.100975

**Published:** 2020-03-11

**Authors:** Shuangshuang Zhao, Xuemeng Shi, Yue Zhang, Zeyu Wen, Jinping Cai, Wei Gao, Jiayi Xu, Yifei Zheng, Baohua Ji, Yanqin Cui, Kun Shi, Yanjun Liu, Hui Li, Yaming Jiu

**Affiliations:** 1 The Joint Program in Infection and Immunity, Guangzhou Women and Children's Medical Center, Guangzhou Medical University, Guangzhou 510623; Institut Pasteur of Shanghai, Chinese Academy of Sciences, Shanghai 200031, China; 2 The Center for Microbes, Development and Health, Key Laboratory of Molecular Virology and Immunology, Institut Pasteur of Shanghai, Chinese Academy of Sciences, Shanghai 200031, China; 3 University of Chinese Academy of Sciences, Yuquan Road No. 19(A), Shijingshan District, Beijing 100049, China; 4 Institute of Applied Mechanics, Zhejiang University, Hangzhou 310027, China; 5 Shanghai Institute of Cardiovascular Diseases, and Institutes of Biomedical Sciences, Zhongshan Hospital, Fudan University, Shanghai 200032, China; 6 Suzhou Institute of Biomedical Engineering and Technology, Chinese Academy of Sciences, Suzhou 215163, China

**Keywords:** Biological Sciences, Cell Biology, Functional Aspects of Cell Biology

## Abstract

Actin stress fibers guide cell migration and morphogenesis. During centripetal flow, actin transverse arcs fuse accompanied by the formation of myosin II stacks to generate mechanosensitive actomyosin bundles. However, whether myosin II stack formation plays a role in cell mechano-sensing has remained elusive. Myosin-18B is a “glue” molecule for assembling myosin II stacks. By examining actin networks and traction forces, we find that cells abolishing myosin-18B resemble Ca^2+^∕calmodulin-dependent kinase kinase 2 (CaMKK2)-defective cells. Inhibition of CaMKK2 activity reverses the strong actin network to thin filaments in myosin-18B-overexpressing cells. Moreover, AMP-activated protein kinase (AMPK) activation is able to relieve the thin stress fibers by myosin-18B knockout. Importantly, lack of myosin-18B compromises AMPK-vasodilator-stimulated phosphoprotein and RhoA-myosin signaling, thereby leading to defective persistent migration, which can be rescued only by full-length and C-extension-less myosin-18B. Together, these results reveal a critical role of myosin-18B in the mechanosensitive regulation of migrating cells.

## Highlights

• Myosin-18B knockout cells resemble cells dampening mechano-sensing signaling pathway
• Myosin-18B depletion decreases the phosphorylation level of AMPK-VASP and MLC
• Myosin-18B knockout cells show compromised persistent migration
• The N-extension and coiled-coil domain of myosin-18B is indispensable in cell migration

## Graphical Abstract

**Figure:**
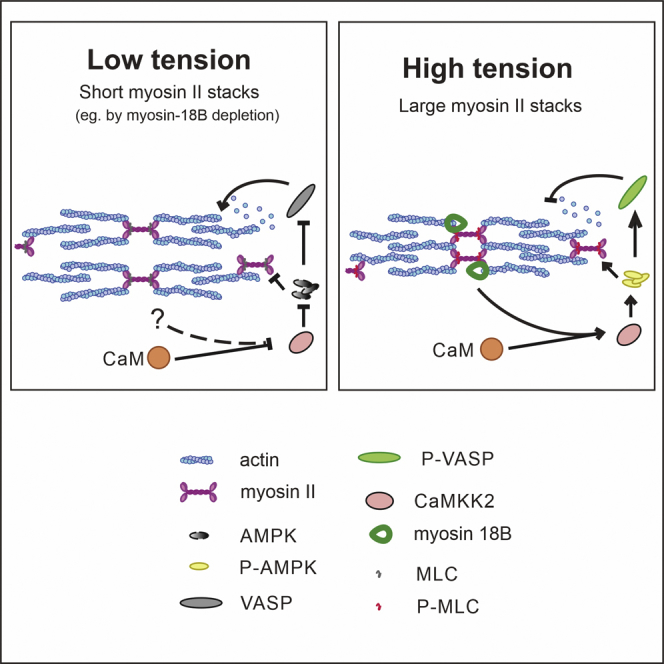

## Introduction

The ability of cells to do persistent migration, to exert forces to the environment, and to conduct mechano-transduction depends on the actin cytoskeleton (Burridge and Wittchen, 2013, Kassianidou and Kumar, 2015, Tojkander et al., 2012). Many non-muscle cell types harbor contractile actomyosin bundles composed of bipolar arrays of actin and non-muscle myosin II collectively, which are called *stress fibers*. Based on protein compositions and associations with focal adhesions, stress fibers can be divided into three subcategories (Naumanen et al., 2008, Small et al., 1998). They are non-contractile “dorsal stress fibers,” thin “transverse arcs” that undergo retrograde flow toward the cell center, and “ventral stress fibers” that are thick actomyosin bundles generated through coalescence of multiple thin transverse arcs during the centripetal flow. Moreover, the ventral stress fibers represent the major force-sensing and force-generating actomyosin bundles in migrating cells (Burnette et al., 2011, Hotulainen and Lappalainen, 2006, Tee et al., 2015, Tojkander et al., 2015).

The fusion of transverse arcs and consequent formation of ventral stress fibers are accompanied by an increased contractile force (Soine et al., 2015, Tojkander et al., 2015). It was suggested that long-range attractive forces exist between individual myosin II filaments during the stack formation. Thus, transverse arc fusion and myosin II stack formation always coincide during the formation of stress fibers (Beach et al., 2017, Burnette et al., 2014, Fenix et al., 2016, Hu et al., 2017). The assembly and alignment of actomyosin bundles are under precise mechanosensitive control. Their maturation requires mechanosensitive influx of Ca^2+^ and activation of downstream Ca^2+^∕calmodulin-dependent kinase kinase 2 (CaMKK2)-AMP-activated protein kinase (AMPK)-AMPK-vasodilator-stimulated phosphoprotein (VASP) signaling cascade, at least in human osteosarcoma cells (Tojkander et al., 2015, Tojkander et al., 2018). However, due to lack of specific methods to inhibit myosin II stack formation, the function of myosin II stacks has not been completely studied, such as whether integrated myosin II stack formation involves in the regulation of mechanosensitive assembly of contractile stress fibers.

Myosin-18B, a class XVIII unconventional myosin, was originally identified as a tumor suppressor (Nishioka et al., 2002). Subsequently, increasing evidences show that myosin-18B gene mutations and its altered expression levels are involved in the progression of various cancer types including lung, colorectal, and ovarian cancer, as well as in cardiomyopathy and muscle weakness in humans, mice, and zebrafish (Ajima et al., 2008, Alazami et al., 2015, Berger et al., 2017, Gurung et al., 2017, Malfatti et al., 2015, Nakano et al., 2005, Nishioka et al., 2002, Yanaihara et al., 2004). We have identified that in human migrating osteosarcoma cells, depletion of myosin-18B specifically inhibits myosin II stack formation and consequent contractile actin stress fiber maturation (Jiu et al., 2019). Therefore, myosin-18B provides an efficient way to study the physiological significance of myosin II stack formation. In this study, we demonstrate that myosin-18B is critical for mechanosensitive CaMKK2-AMPK-VASP signaling cascade of contractile actin stress fibers.

## Results

### Depletion of Myosin-18B Leads to Thin Stress Fibers and less Cell-Mediated Forces, Similar to the Effects Caused by Compromised CaMKK2 Activity

**Figure 1:**
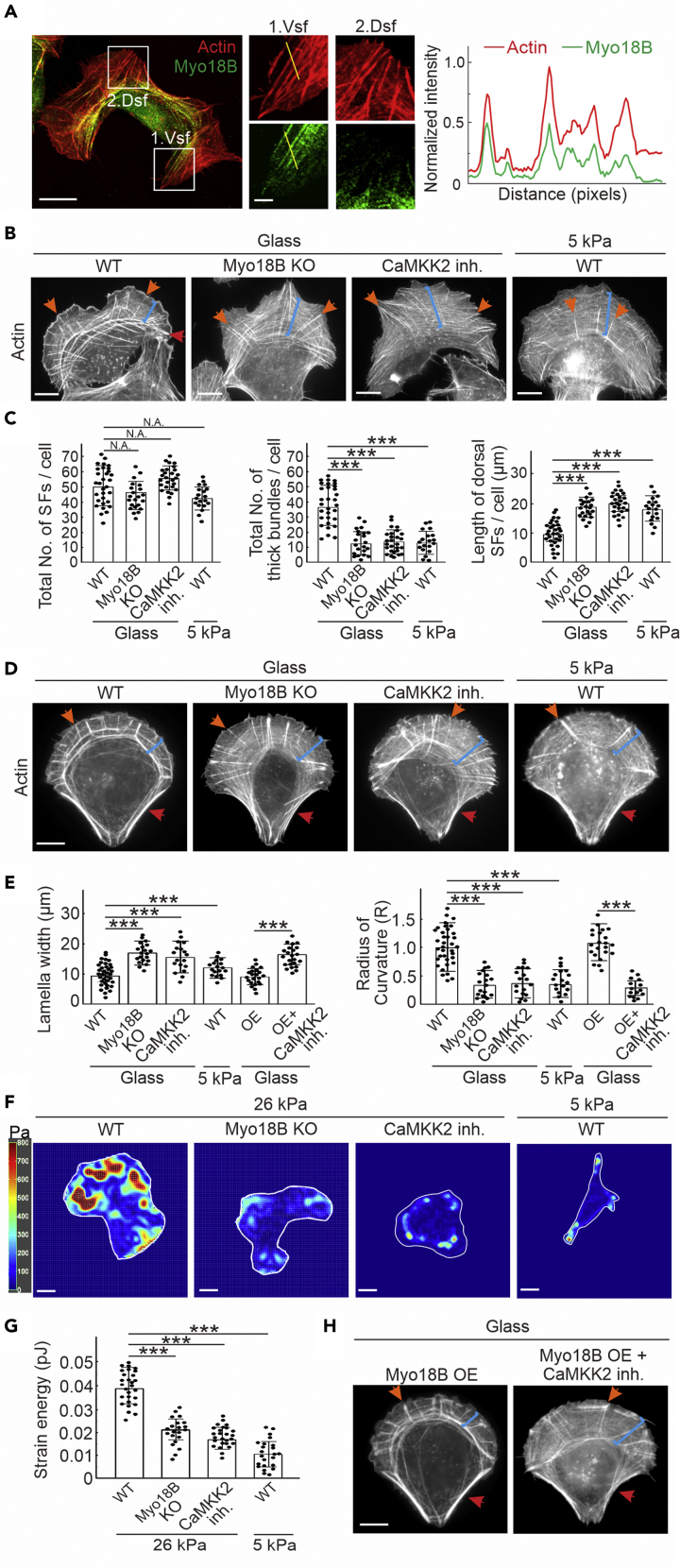
Myosin-18B Knockout Cells Resemble the Cells with Mechanosensitive Defects (A) Left panel shows the representative images of wild-type cells stained with phalloidin and myosin-18B antibody to visualize actin filaments and endogenous myosin-18B. Magnified images (corresponding to the white boxes) display myosin-18B distributions in wild-type cells. Scale bars, 10 and 2 μm in image and magnified images, respectively. Vsf, ventral stress fiber; Dsf, dorsal stress fiber. Right panel shows the line profiles along the yellow line in white box 1, demonstrating the colocalization of endogenous myosin-18B and F-actin at the ends of ventral stress fibers. (B and C) Representative images of actin filaments visualized by phalloidin (B) and quantification of the total numbers of stress fibers and thick bundles and the length of dorsal stress fibers (C) in wild-type cells grown on glass cover slips (n = 31) or softer 5-kPa substrate (n = 19), and myosin-18B knockout (n = 22) and CaMKK2 inhibitor-treated cells (n = 27) grown on glass. (D) Wild-type, myosin-18B knockout and CaMKK2 inhibitor-treated cells grown on crossbow-shaped micropatterns and stained with phalloidin. Scale bar, 10 μm in (B and D). In (B) and (D), orange arrows, dorsal stress fiber; blue brackets, transverse arcs; red arrows, ventral stress fiber. (E) Based on the calculated lamella width and radius of curvature, the ventral stress fibers of myosin-18B-depleted (n = 20), CaMKK2 inhibitor-treated cells grown on glass cover slips (n = 18) and wild-type cells grown on soft substrate (n = 18) are significantly less contractile compared with the wild-type cells grown on glass cover slips (n = 36). The lamella width and radius of curvature are also measured in myosin-18B overexpression cells (n = 22) and further CaMKK2 inhibition-treated cells (n = 23). Values obtained from wild-type cells are normalized to 1. (F and G) (F) Representative traction force maps and (G) quantification of cell-exerted strain energy, which indicates cell-exerted traction forces of wild-type (n = 28 for 26 kPa, n = 23 for 5 kPa), myosin-18B knockout (n = 21), and CaMKK2 inhibitor-treated cells (n = 25). Scale bar, 10 μm. (H) The cells with myosin-18B overexpression are treated with CaMKK2 inhibitor. Scale bar, 10 μm. Quantification data are represented as mean ± SEM. ∗∗∗p < 0.001 (Mann-Whitney-Wilcoxon rank-sum test).

Myosin-18B plays a critical role in maintaining higher-order myosin II stack structures for generation of mature contractile actomyosin bundles (Jiu et al., 2019). Aligning with previous experiments, here we found that the endogenous myosin-18B was dominantly localized to contractile actomyosin bundles, including ventral stress fibers and laterally fused transverse arcs (nuclear-approximate portion of arcs), but not to the non-contractile dorsal stress fibers (Figure 1A). It is to be noted that filamentous myosin-18B did not reach the distal end of ventral stress fiber-associated focal adhesion sites (Figure 1A). In addition, abolishing myosin-18B did not alter the stress fiber-associated expression of myosin IIA, suggesting its specific role in myosin II stack formation (Figure S1A).

Cells with myosin-18B depletion displayed abnormal actin network phenotype, characterized by elongated dorsal stress fibers, thinner transverse arcs, and lack of thick ventral stress fibers (Figure 1B). Interestingly, cells with inhibition of CaMKK2, a newly identified kinase involved in regulating the mechano-sensing signaling cascade in osteosarcoma cells, shared the similar actin network phenotype (Tojkander et al., 2018). In addition, cells grown on compliant matrix (5 kPa) failed to assemble thick contractile ventral stress fibers and thus resembled myosin-18B-deficient cells (Figure 1B). The total number of stress fibers and thick actin filament bundles were calculated by ImageJ with two distinct optimized parameter settings. The quantification confirmed that there were significant but similar decrease in levels of thick bundles in myosin-18B depletion and CaMKK2 inhibition cells and cells grown on softer substrates, whereas the total amount of filaments remained comparable (Figures 1C, S1B, and S1C). In addition, the elongated dorsal stress fiber phenotype was confirmed by quantifying their lengths (Figure 1C). To allow more precise analysis of stress fiber phenotype, cells were plated on crossbow-shaped micropatterns, where they obtained nearly identical shapes and displayed characteristic organization of the stress fiber network (Jiu et al., 2015). It was clear that the longer dorsal stress fibers and thinner transverse arcs were observed and found to be indistinguishable in myosin-18B depletion and CaMKK2 inhibition cells and cells grown on soft matrix (Figure 1D). This abnormal actin network was significantly different from that of wild-type cells cultured on stiff micropatterns with regard to the width of lamella and the radius of curvature (Figure 1E). The stress fiber phenotype was further verified by RNA silencing and could be rescued by reintroducing myosin-18B through transient transfection of myosin-18B-GFP into myosin-18B-silenced cells (Figures S1D–S1F). Moreover, traction force microscopy revealed that cells with myosin-18B depletion or CaMKK2 inhibition and cells cultured on softer matrix displayed similar defects in exerting forces to the substratum, leading to an apparent decrease of the strain energy and mild decrease of the ratio of traction moment matrix α. These two parameters indicate the integrated measure of cell traction and cellular distribution of the traction, respectively (Figures 1F, 1G, and S1G). Thus the actin organization phenotypes of defective myosin II stack formation caused by myosin-18B depletion and of compromised mechano-sensing pathway caused by CaMKK2 inhibition were well aligned. These results indicated that there might be a potential correlation between myosin-18B-modulated myosin II stack formation and cell mechano-sensing regulation.

Overexpression of myosin-18B resulted in moderate to strong actin network, which was comparable with wild-type cells (Figures 1E and 1H). To clarify the relationship and regulatory contribution between CaMKK2 and myosin-18B, we applied CaMKK2 inhibitor to myosin-18B-overexpressing cells grown on micropatterns and found that the contractile stress fibers became thinner followed by wider lamella (Figures 1E and 1H), suggesting that myosin-18B was indeed involved in the regulation of CaMKK2-modulated mechano-sensing.

### Cells Depleted of Myosin-18B Show Indistinguishable Actin-Associated Phenotypes as Wild-Type Cells Grown on Soft Matrix

**Figure 2:**
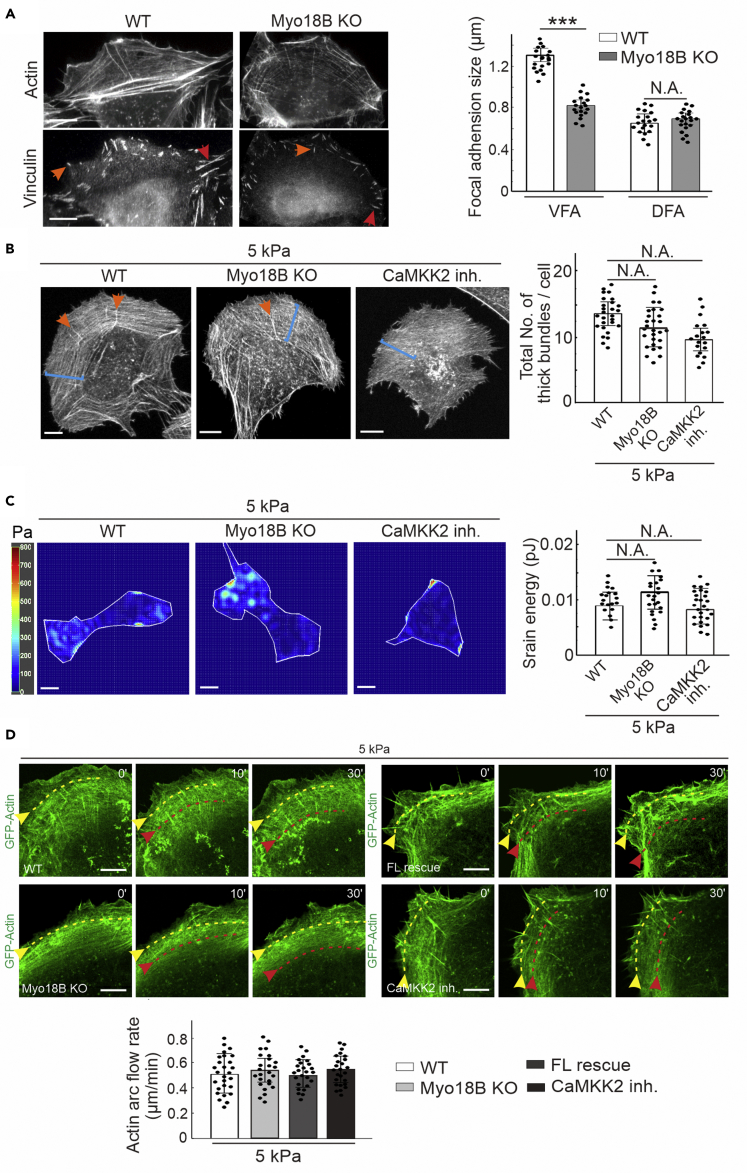
Wild-Type and Myosin-18B-Depleted Cells Show Similar Actin-Associated Phenotypes When Cells Are Grown on Soft Substrates (A) The representative imaging and quantification of ventral- and dorsal-associated focal adhesions in wild-type (n = 19 for vFA, n = 20 for dFA) and myosin-18B knockout (n = 19 for vFA, n = 21 for dFA) cells. vFA (red arrows), ventral associated focal adhesion; dFA (orange arrows), dorsal associated focal adhesion. (B) Representative images of actin filaments and quantification of the total numbers of thick bundle in wild type (n = 27), myosin-18B knockout (n = 27) and CaMKK2 inhibition (n = 19) cells grown on softer matrix (5 kPa). orange arrows, dorsal stress fiber; blue brackets, transverse arcs. (C) Representative traction force maps and quantification of cell-exerted strain energy of wild-type (n = 19), myosin-18B knockout (n = 23), and CaMKK2 inhibitor-treated cells (n = 27) grown on softer matrix (5 kPa). Scale bars, 10 μm in (A–C). (D) Representative examples (corresponding to Video S1, S2, S3, and S4) and quantified centripetal flow rates of transverse arcs in wild-type cells (n = 27), myosin-18B knockout cells (n = 25), full-length (FL) myosin-18B rescue cells (n = 25), and CaMKK2 inhibitor treated cells (n = 25) expressing GFP-actin grown on softer matrix (5 kPa). Yellow arrows, the positions of the observed arcs in the beginning of the videos; red arrows, the positions of the same arcs in subsequent time-lapse images. Scale bar, 5 μm. Quantification data are represented as mean ± SEM. ∗∗∗p < 0.001 (Mann-Whitney-Wilcoxon rank-sum test).

Focal adhesions are mechanosensitive structures (Iskratsch et al., 2014). We found that there were smaller mature focal adhesions in myosin-18B-depleted cells grown on glass cover slips compared with wild-type cells. The size of ventral stress fiber-associated focal adhesions was dramatically reduced in myosin-18B-depleted cells, whereas dorsal stress fiber-associated focal adhesions remained a comparable size (Figure 2A). This result suggested that myosin-18B specifically regulated the mechanosensitive ventral stress fibers, which were equipped with contractile ability.

We further studied whether myosin-18B was critical for proper actomyosin network and traction forces when cells were cultured on softer matrix. Different from growth on rigid substrates, all the cells grown on softer matrix (5 kPa) including wild-type, myosin-18B knockout and CaMKK2-inhibited cells shared similar thin actin network architectures and lost the thick actin bundles (Figures 2B and S2A). Moreover, the effect of myosin-18B depletion on the levels of strain energy detected by traction force microscopy was negligible when the cells were plated on 5-kPa matrix, and similar weak forces were observed in cells treated with CaMKK2 inhibitor on the same soft substrates (Figure 2C).

In wild-type cells, the transverse arcs first become visible near the leading edge of the cell. The consequent assembly of thick ventral stress fibers occurs through fusion of the arcs during their centripetal flow (Tojkander et al., 2015, Jiu et al., 2019). By live cell imaging expressing GFP-actin, we visualized the centripetal flow in real time. In line with early study (Jiu et al., 2019), myosin-18B depletion led to faster actin flow velocity when the cells were grown on glass cover slips, thereby causing less coalescence of thin transverse arcs (Figure S2B). However, the averaged rates of actin centripetal flow showed no significant difference in all the cells grown on softer 5-kPa matrix, including myosin-18B knockout, wild-type, full-length re-expression and CaMKK2 inhibition cells (Figures 2D and Video S1, S2, S3, and S4). Together, these data provided evidence that myosin-18B played a critical role in the force-producing maturation of ventral stress fibers, without affecting non-contractile actin structures.

Video S1 Time-Lapse Imaging of GFP-Actin-Expressing Wild-Type Cells Grown on 5-kPa Matrix, Related to Figure 2 Duration of the video was 30 min, and the recording time interval is 10 s/frame.

Video S2 Time-Lapse Imaging of GFP-Actin-Expressing Myosin-18B Knockout Cells Grown on 5-kPa Matrix, Related to Figure 2 Duration of the video was 30 min, and the recording time interval is 10 s/frame.

Video S3 Time-Lapse Imaging of GFP-Actin-Expressing Myosin-18B Rescue Cells Grown on 5-kPa Matrix, Related to Figure 2 Duration of the video was 30 min, and the recording time interval is 10 s/frame.

Video S4 Time-Lapse Imaging of GFP-Actin-Expressing CaMKK2 Inhibition Cells Grown on 5-kPa Matrix, Related to Figure 2 Duration of the video was 30 min, and the recording time interval is 10 s/frame.

### Myosin-18B Depletion Compromises Mechanosensitive Tension-Induced AMPK-Mediated Phosphorylation of VASP

**Figure 3:**
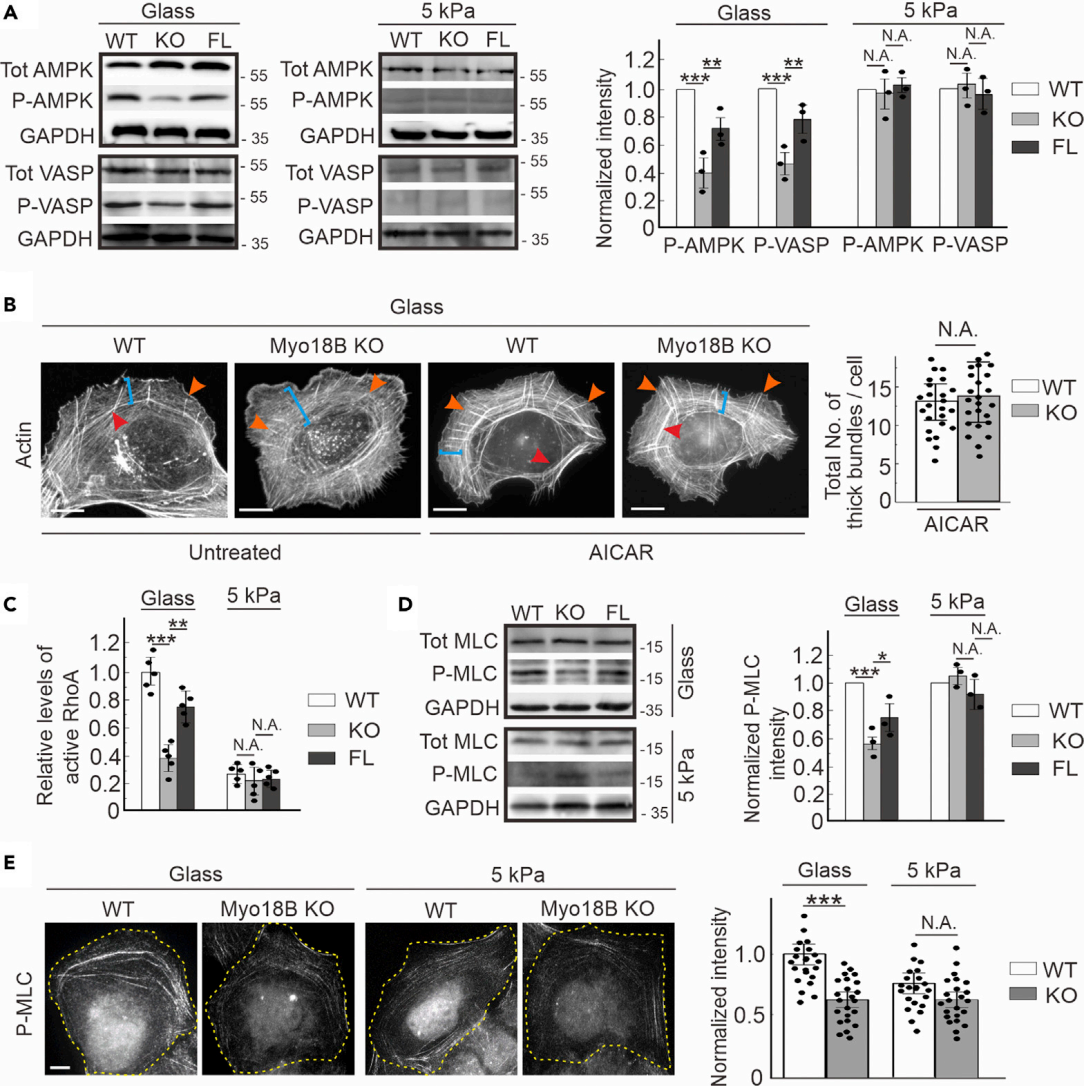
Myosin-18B Depletion Compromises Mechanosensitive AMPK-VASP Phosphorylation (A) Western blot analysis of endogenous total AMPK, VASP, phospho-AMPK, and phospho-VASP levels in the lysates of wild-type, myosin-18B knockout, and full-length rescue (FL) cells grown on glass cover slips and soft substrates (5 kPa). The quantifications are from three independent experiments. GAPDH is probed for equal sample loading. (B) Representative images of actin filaments visualized by phalloidin, and quantification of total number of thick bundles in wild-type (n = 24) and myosin-18B knockout cells (n = 24) treated with AICAR on glass cover slips. Orange arrows, dorsal stress fiber; blue brackets, transverse arcs; red arrows, ventral stress fiber. Scale bar, 10 μm. (C) The level of active RhoA detected by G-Lisa in wild-type, myosin-18B knockout, and full-length rescue cells grown on glass cover slips and soft substrates (5 kPa). Quantification is based on five independent experiments. (D) Western blot analysis of endogenous total MLC and phospho-MLC levels in the lysates of wild-type, myosin-18B knockout, and full-length rescue cells grown on glass cover slips and soft substrates (5 kPa). (E) Representative images of active MLC visualized by staining with phospho-MLC antibody in wild-type and myosin-18B knockout cells cultured on glass and 5-kPa matrix. Scale bar, 10 μm. Quantification data are represented as mean ± SEM. Values obtained from wild-type cells are normalized to 1 in (A, C, D, and E). ∗p < 0.05, ∗∗p < 0.01, ∗∗∗p < 0.001 (unpaired t test).

Phosphorylation of AMPK at Thr172 is indicative of its activation, and phosphorylation of VASP at the AMPK targeting site Ser239 inhibits actin vectorial polymerization activity at focal adhesion (Benz et al., 2009). Previous work indicates that the calcium-stimulated CaMKK2-AMPK-VASP pathway regulates the mechanosensitive assembly of contractile actin stress fibers in human osteosarcoma cells (Tojkander et al., 2015, Tojkander et al., 2018). We thus examined the levels of AMPK and VASP in myosin-18B knockout cells, and the results exhibited significantly lower phosphorylation levels of AMPK and VASP compared with wild-type cells, whereas reintroducing myosin-18B to gene knockout cells partially restored these effects. Nevertheless, the total protein levels of AMPK and VASP remained unaffected (Figure 3A). As a consequence, actin vectorial elongation was promoted and ventral focal adhesion was thus less matured and displayed as smaller size as we observed (Figure 2A). Moreover, when all above-mentioned cells were cultured on a softer substrate (5 kPa), they display dramatically diminished phosphorylation levels of both AMPK and VASP (Figure 3A), suggesting that the soft matrix is (at least) one of the extracellular stimulations leading to compromised AMPK-VASP pathway, despite cells with relevant endogenous gene knockout. To exclude the possibility that cell growth status was affected by myosin-18B, we performed MTT and bromodeoxyuridine assays, which confirmed that the viability and proliferation of the cells were not apparently influenced by myosin-18B depletion in both stiff and soft growing matrix (Figures S3A and S3B).

Active level of AMPK is regulated in a tension-dependent manner, and this is essential for the maturation of force-producing actomyosin bundles (Tojkander et al., 2015). Thus, we increased the active level of AMPK by applying its activator, AICAR, to both wild-type and myosin-18B knockout cells cultured on glass cover slips. Western blot experiment showed that activation of AMPK by AICAR led to an increased phosphorylation of VASP (Figure S3C). Furthermore, upon AICAR treatment, wild-type cells displayed conventional strong dorsal, arc, and ventral stress fibers (Figure 3B). It was worth noting that the phenotype of thinner stress fibers was apparently relieved and assembled into thick bundles by AICAR treatment in myosin-18B knockout cells (Figures 3B and S3D). This result indicated that the mechano-sensing pathway promoted by active AMPK can bypass the actin stress fiber defects caused by myosin-18B depletion.

It is to be noted that the small GTPase RhoA regulates myosin light chain phosphorylation and activities of several actin-binding proteins to promote stress fiber contractility and assembly (Guilluy et al., 2011a, Guilluy et al., 2011b, Lessey et al., 2012). We consequently examined the levels of active RhoA by using a well-established G-LISA assay (Jiu et al., 2017). Absence of myosin-18B significantly decreased the level of active GTP-bound RhoA (Figure 3C), indicating the synergy coordination of actin filaments' assembly and myosin II stack function. Wild-type and myosin-18B knockout cells were blotted and stained with an antibody detecting phosphorylated (Thr18/Ser19) myosin light chain (P-MLC), showing that myosin-18B abolishment dramatically reduced both P-MLC level and fluorescence intensity. This phenotype can be partially rescued when myosin-18B was reintroduced (Figures 3D and 3E). It was interesting to note that myosin-18B-abolished cells grown on either glass cover slips or soft substrates (5 kPa) had comparable decrease of P-MLC intensity when compared with wild-type cells (Figure 3E), further suggesting the correlation between myosin-18B and tension-induced cell mechano-sensing.

### Myosin-18B Depletion Reduces the Cell-Directed Migration

**Figure 4:**
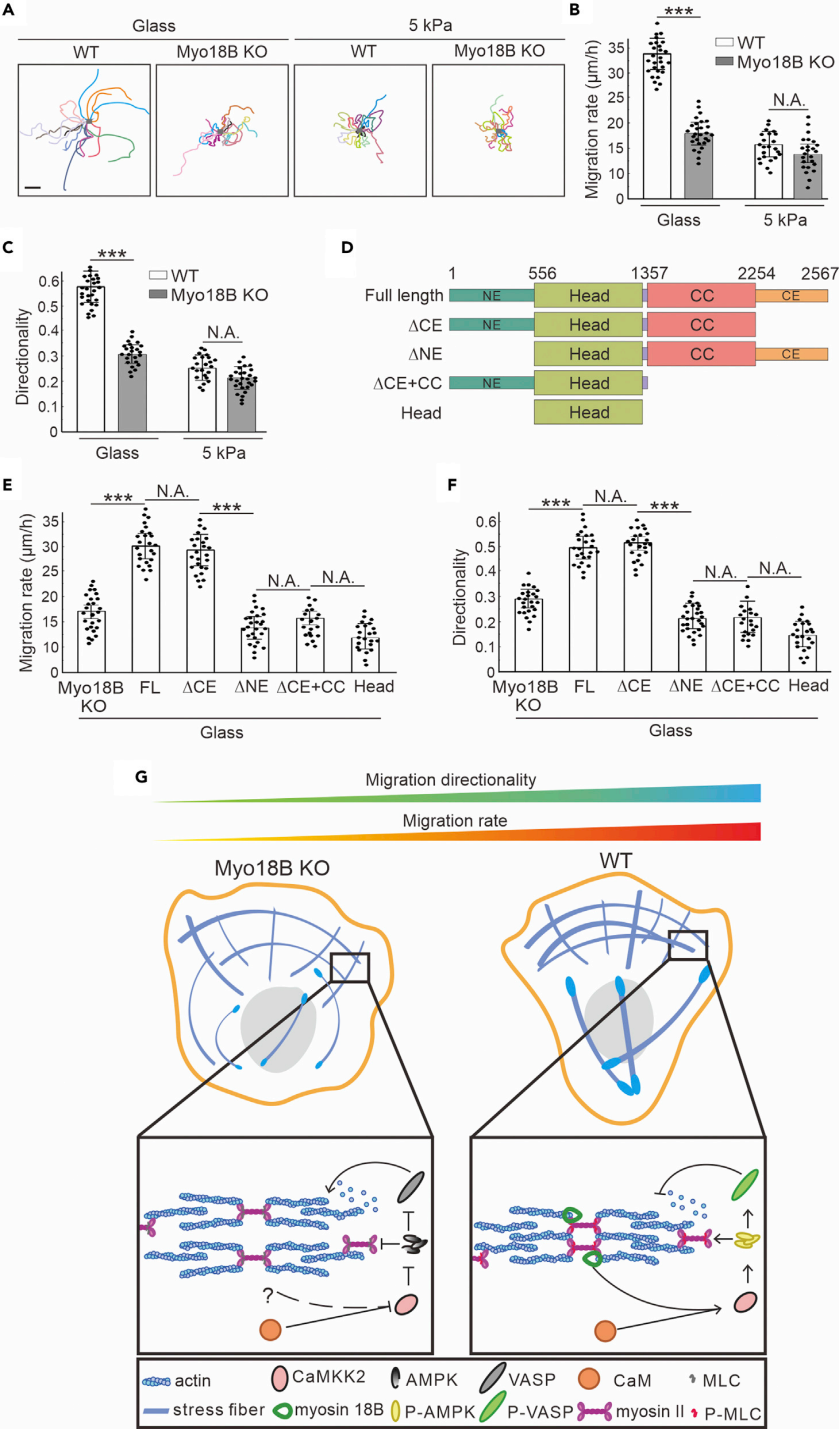
Myosin-18B Deletion Inhibits the Directed Migration of Cells on Rigid Substrates (A) Representative images of the moving trajectory of individual wild-type or myosin-18B knockout cell seeded on fibronectin-coated glass or 5-kPa matrix. Scale bar, 40 μm. (B and C) Quantification of the (B) migration rate and (C) directionality of wild-type (n = 27 for glass, n = 23 for 5 kPa) and myosin-18B knockout cells (n = 24 for glass, n = 28 for 5 kPa). (D) Truncated myosin-18B constructs used in the rescue experiments. (E and F) Quantification of the (E) migration rate and (F) directionality of different myosin-18B constructs expressed in myosin-18B knockout cells. n = 25 for myosin-18B-FL-GFP, n = 25 for ΔCE-myosin-18B, n = 27 for ΔNE-myosin-18B, n = 21 for ΔCE + CC-myosin-18B, and n = 23 for head-myosin-18B transfected cells were used for quantification. Data are represented as mean ± SEM. ∗∗∗p < 0.001 (Student's t test). (G) A working model for the regulation of myosin-18B in the mechanosensitive CaMKK2-AMPK-VASP/MLC signaling cascade and directed cell migration.

Myosin-18B depletion cells display more roundish morphology and dramatically reduced movement (Jiu et al., 2019). However, whether myosin-18B regulates other motility properties, such as migration direction persistency, or not remain elusive. We tracked the moving trajectory of individual cell seeded on fibronectin-coated glass cover slips and found that there was a significant reduction of the migration rate and the directionality index (Figures 4A–4C). However, when cells were switched to softer matrix (5 kPa), both wild-type and myosin-18B knockouts cells lost the dynamic mobility and relatively consistent direction persistency and showed no significant difference with each other (Figures 4A–4C).

To explore the contributions of head and coiled-coil domains as well as the N- and C-terminal extensions of myosin-18B, GFP-tagged mutant proteins were expressed in myosin-18B knockout cells (Figure 4D) and tested for their ability to rescue the characteristic defects in cell migration. Only cells expressing intermediate levels of GFP fluorescence intensity were selected for analysis. Among four truncations, the protein lacking the C-terminal extension displayed comparable cell motility characteristics as the full-length rescue cells (Figures 4E and 4F). Thus, the N-terminal extension, head domain, and coiled-coil domain are critical for cellular function of myosin-18B, whereas the C-terminal extension had only a minor role.

## Discussion

Myosin-18B supports the lateral association of individual myosin II filaments or stacks with each other (Jiu et al., 2019). In this study, we provide evidence that in addition to the role as “molecular glue,” myosin-18B is also involved in regulating the mechanosensitive CaMKK2-AMPK-VASP/MLC signaling cascade, which is related to the persistent migration of the cells (Figure 4G). Owing to the fact that myosin-18B depletion does not result in complete inhibition of myosin II stack formation (Jiu et al., 2019), there might be other unidentified regulatory factors that are likely to contribute to the integrity of the actomyosin bundles and related mechanosensitive pathways, such as tropomyosin and α-actinin.

The defective mechanosensitive signaling is also a possible reason for the various cellular phenotypes caused by myosin-18B depletion because the remaining thin arcs are still contractile in myosin-18B knockout cells. We speculate the possibility that changing the compactivity of actomyosin bundles by alignment of myosin II stacks could regulate the mechano-sensing ability of cells. Moreover, our data propose that, at least in osteosarcoma cells, contractile stress fibers are not able to maintain or become thicker upon tension unless there is expression of myosin-18B. Taken together, our study provides important new insight into the physiological importance of the higher-order myosin II structures in cells and uncovers its association with cell mechanics regulation. These findings also provide an explanation for the association of myosin-18B mutations with various diseases, including cancers and myopathies.

In the future, it will be interesting to examine the function and underlying mechanisms of myosin-18B-related myosin II stacks upon imposed external forces. In addition, because many cell types including epithelial cells can assemble peripheral actomyosin bundles resembling ventral stress fibers, it will be equally interesting to explore the physiological function of myosin II stacks in non-motile cells by taking advantage of myosin-18B as a tool. In addition, it will be important to reveal how the activities of other proteins contribute to myosin II stack formation and consequent mechanosensitive regulation.

### Limitations of the Study

Some limitations to the findings of this study must be acknowledged. First, there are two myosin-18B-related clinical studies up to date (Alazami et al., 2015, Malfatti et al., 2015), which discovered two nonsense mutations of myosin-18B (c.6905C > A:p.S2302∗; c.6496G > T:p.Glu2166∗). Two patients with 6905C > A showed Klippel-Feil anomaly and myopathy phenotypes, and the patient with 6496G > T showed severe nemaline myopathy with cardiomyopathy. However, there is no mechanism study focusing on these two mutations. In our study, despite the fact that we dissected the function of distinct myosin-18B domains, we did not explore whether these two nonsense mutations identified from clinical patients show any potential importance in the mechano-sensing pathway. Because these patients display myopathy-related disease, it would be interesting to explore whether mechano-sensing defects are a possible cause of the disease in muscle system. Second, it would be also interesting to explore whether myosin-18A, the other member of the myosin-18 family, is involved in the mechano-sensing pathway in this study. Third, due to that there is high expression of myosin-18B in muscle system, e.g., cardiomyocytes. It would be interesting to study myosin-18B-related mechano-sensing pathway in the context of differentiation and developmental regulation of cardiomyocytes. These subject matters have been left untouched in this article but deserve future investigation.

## Methods

All methods can be found in the accompanying Transparent Methods supplemental file.

## Supplemental Information

Document S1. Transparent Methods and Figures S1–S3
