# Supplementary material for: Myosin-18B Promotes Mechanosensitive CaMKK2-AMPK-VASP Regulation of Contractile Actin Stress Fibers
Source: iScience. 2020 Mar 11;23(4):100975. doi: 10.1016/j.isci.2020.100975 (PMC7109629; doi:10.1016/j.isci.2020.100975)
Supplement: Document S1. Transparent Methods and Figures S1–S3 [file mmc1.pdf]

iScience, Volume *23*

Supplemental Information

Myosin-18B Promotes Mechanosensitive CaMKK2-AMPK-VASP Regulation of Contractile Actin Stress Fibers

Shuangshuang Zhao, Xuemeng Shi, Yue Zhang, Zeyu Wen, Jinping Cai, Wei Gao, Jiayi Xu, Yifei Zheng, Baohua Ji, Yanqin Cui, Kun Shi, Yanjun Liu, Hui Li, and Yaming Jiu

Supplemental Information

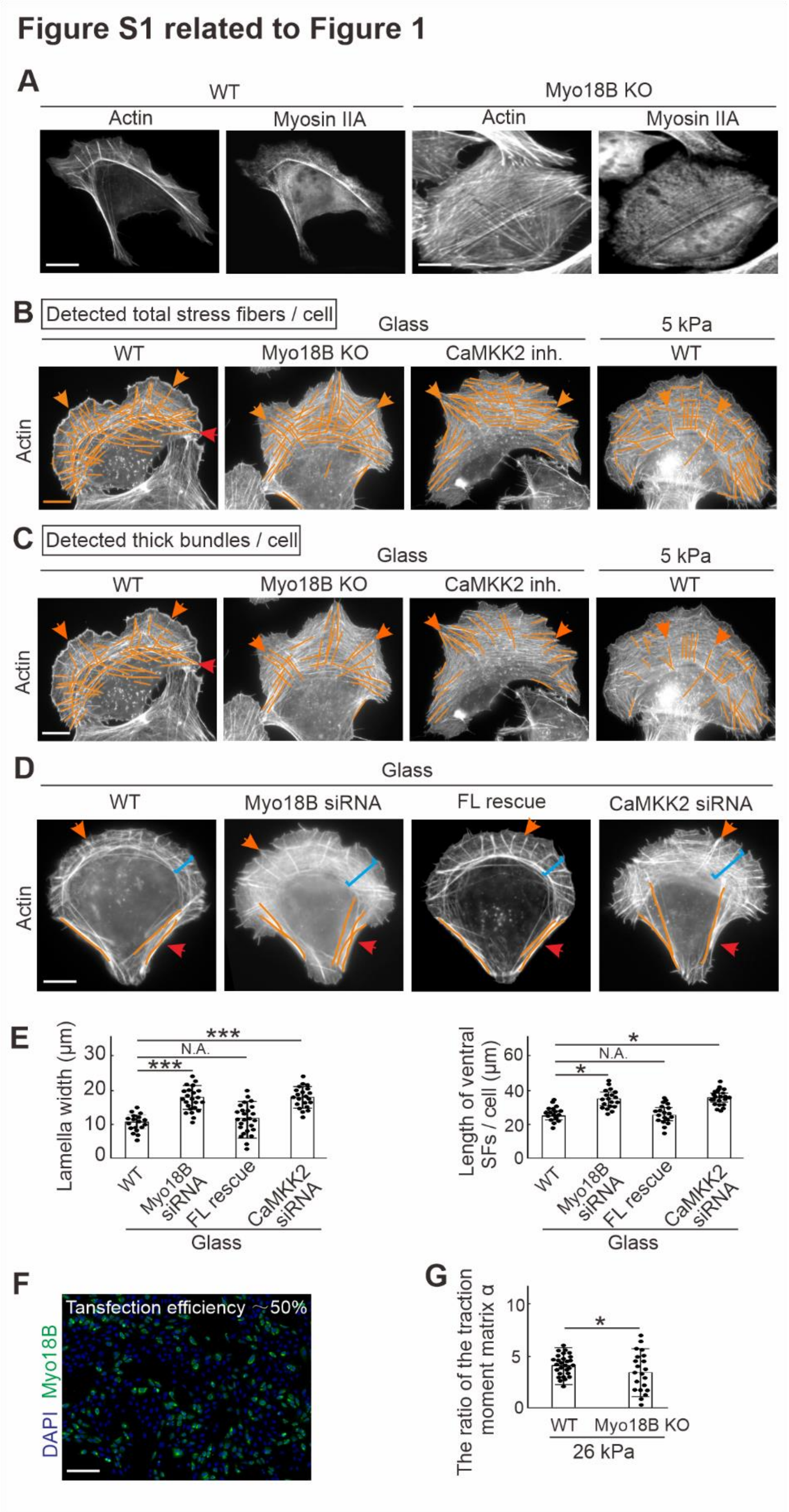

Figure S1 Depletion of myosin-18B and inhibition of CaMKK2 share similar actin network defects, Related to Figure 1. (A) Representative images of actin and myosin

II in wild type and myosin-18B knockout cells. (B and C) The detected total numbers of stress fibers (B) and thick bundles (C) are done by ‘rigid detection’ plugin of Fiji ImageJ with distinct settings for the representative images in Figure 1B. The scale bar represents 10 μm.   (D and E) Representative examples and quantification of lamella width and length of ventral stress fibers of wild type, myosin-18B and CaMKK2 knockdown, and myosin-18B-reintroducing cells cultured on glass micropatterns. The scale bar represents 10 μm. (F) The representative example to show the transfection efficiency of myosin-18B-GFP to myosin-18B knockout cells. (G) The quantification of the ratio of the traction moment matrix α of wild type (n=28 for 26 kPa), myosin-18B knockout (n=21 for 26 kPa). *p< 0.05, ***p < 0.001 (Student *t* test).

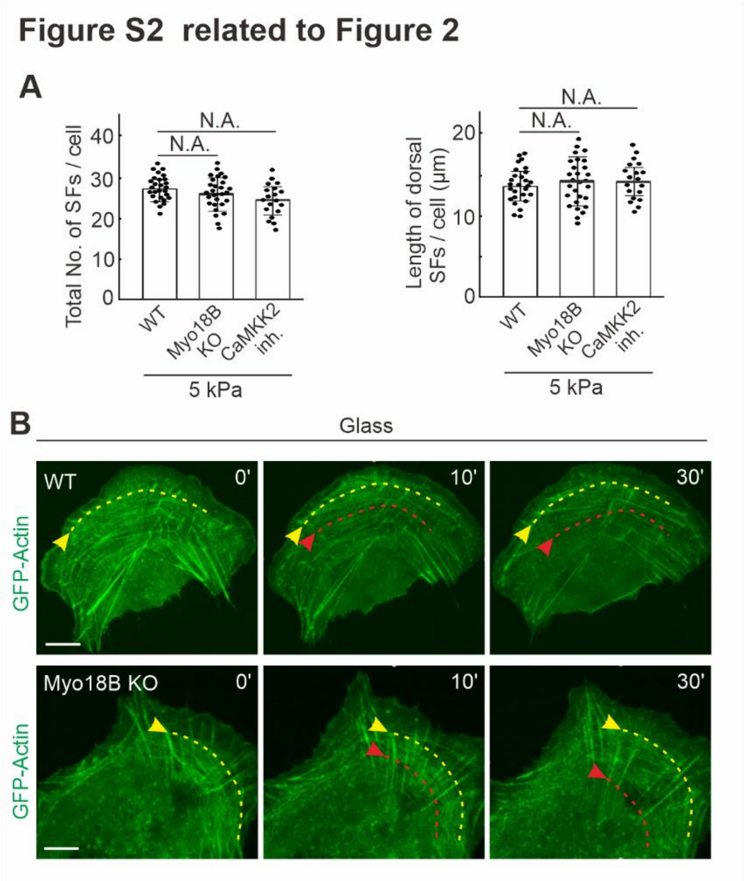

Figure S2 Actin retrograde flow is faster in myosin-18B depletion cells grown on glass, Related to Figure 2. (A) Quantification of the total number of stress fibers and the length of dorsal stress fibers in wild type, myosin-18B knockout and CaMKK2 inhibition cells grown on softer matrix (5 kPa). (B) Representative examples of transverse arc flow in wild type and myosin-18B knockout cells expressing GFP-actin grown on glass cover slips. Yellow arrows, the positions of the observed arcs in the beginning of the movies; red arrows, the positions of the same arcs in subsequent time-lapse images. The scale bar represents 10 µm.

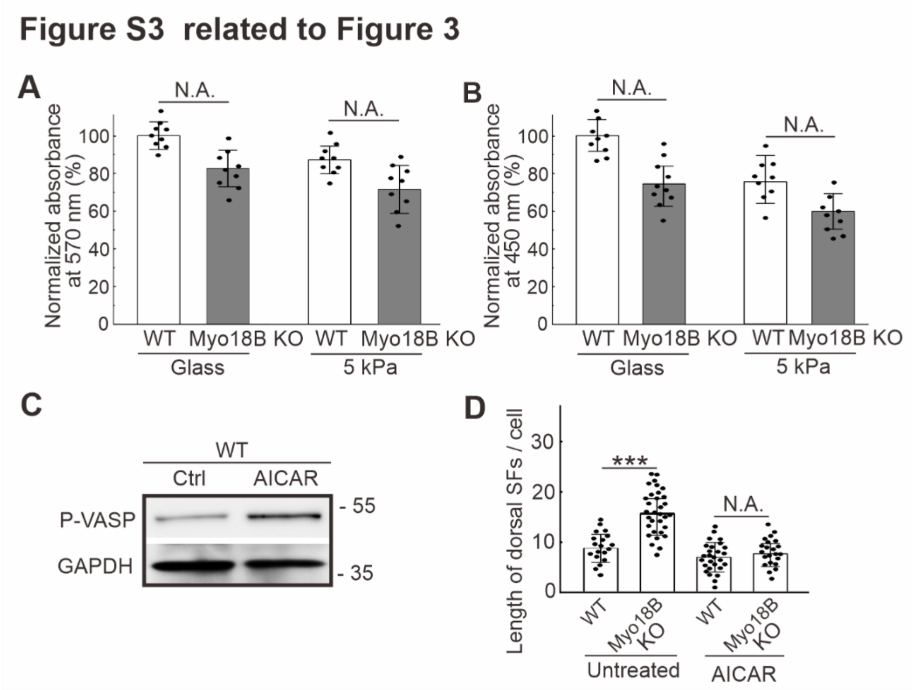

Figure S3 Myosin-18B depletion and CaMKK2 inhibition do not affect cell proliferation and viability, Related to Figure 3. (A) Quantification of the absorbance at 570 nm detected via MTT assay in wild type and myosin-18B knockout cells grown on glass cover slips and soft substrates (5 kPa). n=9 from three independent experiments. (B) Quantification of the absorbance at 450 nm detected via BrdU assay in wild type and myosin-18B knockout cells grown on glass cover slips and soft substrates (5 kPa). (C) Western blot analysis of phospho-VASP level upon AICAR treatment in wild type cells. GAPDH is probed for equal sample loading. (D) Quantification of the length of dorsal stress fibers in wild type and myosin-18B knockout cells grown on glass with or without AICAR treatment. ***$p < 0.001$ (unpaired *t* test).

Transparent Methods

Cell culture, transfection and treatment

Human osteosarcoma (U2OS) cells were maintained in high-glucose (4.5 g/l) Dulbecco's modified Eagle's medium (DMEM, BE12-614F; Lonza, Basel, Switzerland), supplemented with 10% Fetal Bovine Serum (10500-064; Gibco, Waltham, MA, USA), 10 U/ml penicillin, 10 μg/ml streptomycin, and 20 mM L-glutamine (10378-016; Gibco) at 37°C in humidified atmosphere with 5% CO_2. Myosin-18B knockout U2OS cells were generated and well verified in our previous work (Jiu et al., 2019), and used in this study. Transient transfections were performed with Fugene HD (Promega, Madison, WI, USA), following the manufacturer's instructions using 3.5:1 Fugene/DNA ratio, and cells were incubated for 24 h before fixation with 4% paraformaldehyde (PFA) in phosphate-buffered saline (PBS). For inhibition of CaMKK2, cells were treated with 10 mM STO-609 (S1318, Sigma-Aldrich) for 3 h. For activation of AMPK, cells were treated with 25 μM AICAR (A9978, Sigma-Aldrich) for 16 h.

Immunofluorescent microscopy

Immunofluorescence (IF) experiments were performed as previously described (Jiu et al., 2017; Lehtimaki et al., 2017). Briefly, cells were fixed with 4% PFA in PBS for 15 min at room temperature (RT), washed three times with 0.2% BSA in Dulbecco's phosphate buffered saline, and permeabilized with 0.1% Triton X-100 in PBS for 5 min. Cells were blocked in 1× Dulbecco PBS supplemented with 0.2% BSA. The following primary antibodies were used for immunofluorescence: myosin-18B rabbit polyclonal antibody (1:50 dilution; LS-C403352, LSBio, Seattle, WA, USA); phospho-myosin light chain 2 (Thr18/Ser19) rabbit polyclonal antibody (1:50 dilution; #3674, Cell Signaling, Beverly, MA, USA). Both primary and secondary antibodies were applied onto cells and incubated at RT for 1 h. Alexa-conjugated phalloidin was added together with primary antibody solutions onto cells. All IF data were obtained with a Leica DM6000B wide-field fluorescence microscope with a HCXPL APO 63×, NA 1.40 oil

objective. For micropattern experiments, the cells were plated on CYTOOchips™ prior to fixation as described previously (Jiu et al., 2019).

Live cell imaging

The cells were plated prior to imaging on 10 μg/ml fibronectin-coated glass bottomed dishes (MatTek Corporation, Ashland, MA, USA). The time-lapse images of GFP-actin for centripetal arc flow were acquired with 3I Marianas imaging system (3I intelligent Imaging Innovations, Denver, CO, USA), consisting of an inverted spinning disk confocal microscope Zeiss Axio Observer Z1 (Zeiss, Dublin, CA, USA) and a Yokogawa CSU-X1 M1 confocal scanner (Tokyo, Japan). Appropriate filters, heated sample environment (+37°C), controlled CO_2, and 63×/1.2 WC-Apochromat Corr objective (Zeiss) were used. The images were acquired via SlideBook 6.0 software (3I intelligent Imaging Innovations), and recorded via Neo sCMOS (Andor Inc., UK) camera. One focal plane was recorded for all time lapse videos.

Focal adhesion data quantification

Vinculin mouse monoclonal antibody (dilution 1:100; V9131, Sigma-Aldrich, St. Louis, MO, USA) was used to mark the focal adhesion (FA), whose lengths were manually quantified with ImageJ from wild type and myosin-18B knockout cells. Cells adhered to several neighboring cells were discarded from the analysis, and only the cells that displayed well phalloidin staining were selected for analysis.

Traction force microscopy

Traction force microscopy was used to measure the contractile forces that cells exerted upon their substrate as previously described (Jiu et al., 2017). Briefly, cells were cultured for 3-8 h on custom-made 35-mm dishes (Matrigen Life Technologies, CA, USA) with fibronectin-coated polyacrylamide gel with either 26- or 5- kPa stiffness. The diameter of 200 nm yellow-green fluorescent (505/515) microspheres were immobilized to the surface of the gel. Images of the cells and of the fluorescent

microspheres directly underneath the cells were acquired during the experiments and after cell detachment with trypsin. By comparing the reference image with the experimental image, we computed the cell-exerted displacement field. From the displacement fields, and manual traces of the cell contours, together with knowledge of substrate stiffness, we computed the traction force fields using the approach of constrained Fourier-transform traction cytometry. From the traction fields, we calculated the strain energy by equation

$$U = \frac{1}{2}\int \boldsymbol{T}(r).\boldsymbol{u}(r)dA$$

It is the total deformation energy produced by the cells through applying the traction on the surface of the substrate, which suggested an integrated measure of cell traction. To measure the distribution of the traction, we calculated the first order moment of the traction by equation

$$M_{ij} = \frac{1}{2}\int \left(x_i T_j + x_j T_i\right)dA$$

which is a two-dimensional matrix. The net moment is given by the trace of the matrix as $\mu = tr(\mathbf{M})$, which is the measure of the contractile strength of cell. And the distribution of the traction can be measured by the ratio of the two principal values of the traction moment matrix as $\alpha = \lambda_{max}/\lambda_{min}$, which can be calculated by solving the eigenvalue problem $M_{ij} - \lambda I_{ij} = 0$ (He et al., 2020).

Western blotting

All cell lysates were prepared by washing the cells once with PBS and scraping them into lysis buffer (50 mM Tris-HCl pH 7.5 150 mM NaCl, 1 mM EDTA, 10% Glycerol, 1% Triton X-100) supplemented with 1 mM PMSF, 10 mM DTT, 40 μg/ml DNase I and 1 μg/ml of leupeptin, pepstatin, and aprotinin. All preparations were conducted at 4°C. Protein concentrations were determined with Bradford reagent (#500-0006, Bio-Rad, Richmond, California, USA). Then western blotting was performed as described previously (Jiu et al., 2019). The following antibodies were used in this assay: phospho-myosin light chain 2 (Thr18/Ser19) rabbit polyclonal antibody (dilution 1:500; #3674, Cell Signaling); myosin light chain mouse monoclonal antibody (dilution 1:1000;

#M4401, Sigma-Aldrich); AMPK rabbit polyclonal antibody (dilution 1:500; SAB4502329, Sigma-Aldrich); phospho-AMPK (Thr172) rabbit polyclonal antibody (dilution 1:500; # 2531S, Cell Signaling); VASP rabbit polyclonal antibody (dilution 1:500; HPA005724, Atlas Antibodies, Stockholm, Sweden); phospho-VASP (Ser239) rabbit polyclonal antibody (dilution 1:500; 16C2, Millipore, Temecula, CA, USA); GAPDH mouse polyclonal antibody (1:1000 dilution; G8795, Sigma-Aldrich).

RhoA activity assay

RhoA activity was determined with a RhoA G-LISA absorbance-based biochemical assay kit (Cytoskeleton Inc., Denver, CO, USA) following the manufacturer's instructions. Briefly, cells were lysed, and binding buffer was added to the cell lysate, followed by an incubation on a RhoA-GTP affinity plate coated with RhoA-GTP-binding protein. The plate was placed on an orbital plate shaker at 400 rpm for 30 min at 4°C. After washing, primary anti-RhoA antibodies (dilution 1:250) and secondary HRP-linked antibodies (dilution 1:62.5) were sequentially added, followed by an incubation on an orbital shaker at 400 rpm for 45 min at RT. Afterwards, HRP-detection reagents were added, and the absorbance was recorded via a plate reader spectrophotometer Enspire (PerkinElmer Company, Waltham, MA, USA).

Fabrication of soft substrate

Gels of polyacrylamide (PAA) of various stiffness were prepared by mixing 40% polyacrylamide and 2% bis-acrylamide solution, as described previously (Pelham and Wang, 1997). Substrate preparation protocols and modulus values were adopted from previously published work (Tse and Engler, 2010). Briefly, the gel solution for desired stiffness was mixed with ammonium persulfate (APS; 1:100) and tetramethylethylenediamine (TEMED; 1:1000) and placed between a hydrophobic glass (octadecyltrichlorosilane treated; 104817, Sigma-Aldrich) and the transparency sheet treated with 3-APTMS (A17714, Alfa Aesar, Ward Hill, MA, USA). Once polymerized, the hydrophobic plate was carefully removed. The gel was conjugated

with sulfo-SANPAH (sulfosuccinimidyl 6 (4-azido-2- nitrophenyl-amino) hexanoate) and incubated with rat tail type I collagen (25 μg/ml) (A1048301, Invitrogen, CA, USA) at 4°C for overnight.

Filament analysis

The total number of stress fibers, as well as the number of thick actin filament bundles in U2OS cells were quantified with ridge detection plugin from Fiji ImageJ. The parameters used for quantifying the total number of stress fibers are: line width 20.0, high contrast 230, low contrast 100, sigma 6.57, low threshold 0.0, and upper threshold 0.34. The parameters used for quantifying the thick bundles are: line width 29.0, high contrast 230, low contrast 87, sigma 8.87, low threshold 0.0, and upper threshold 0.17. Please note that the detection of thick bundles identified some dorsal stress fibers, whereas the defects which supporting the decreased contractile bundles still significant. We manually outlined the dorsal stress fibers in cells on petri dishes and the ventral stress fibers in cells on micropattern chips, and subsequently measured the lengths for quantification. Please note that we made sure that selection of cells etc. for the analyses were completely unbiased by mixing all data together and analyzing blindly by another person.

Cell proliferation assay

Cell proliferation and viability were assessed by MTT and BrdU assay. For MTT assay, in brief, wild type and myosin-18B knockout cells were plated in 96-well plates at 2×10^3 per well, and cultured for 24 h. Then 20 μl of MTT (0.5 mg/ml) was added to each well and the plates were further incubated at 37°C for 4 h. After incubation, medium was removed and 150 μl of DMSO per well was added to dissolve MTT-formazan crystals. Absorbance of each well at 570 nm was recorded with plate reader spectrophotometer Enspire (PerkinElmer). BrdU assay was performed via a BrdU kit (Abcam, Cambridge, UK) in accordance with the manufacturer's instructions. In brief, cells were labeled with BrdU and fixed, followed by an incubation with an anti-BrdU

antibody and HRP-linked IgG. Afterwards, HRP substrate was added, and the absorbance at 450 nm was determined by using plate reader spectrophotometer Enspire (PerkinElmer).

Quantification and statistical analysis

Statistical analyses were performed with Excel (Microsoft, Redmond, WA, USA) and SigmaPlot (Systat Software Inc, San Jose, CA, USA). Sample sizes and the numbers of replications are included in the images. Radius of curvature were calculated with the equation used in previous study (Tojkander et al., 2018). Student's two-sample unpaired *t* test or Mann-Whitney-Wilcoxon rank-sum test was used to assess the statistical difference. $P < 0.05$ was considered to be significant.

Supplemental References

Lehtimaki, J.I., Fenix, A.M., Kotila, T.M., Balistreri, G., Paavolainen, L., Varjosalo, M., Burnette, D.T., and Lappalainen, P. (2017). UNC-45a promotes myosin folding and stress fiber assembly. J Cell Biol 216, 4053-4072.

Pelham, R.J., Jr., and Wang, Y. (1997). Cell locomotion and focal adhesions are regulated by substrate flexibility. Proc Natl Acad Sci U S A 94, 13661-13665.

Tse, J.R., and Engler, A.J. (2010). Preparation of hydrogel substrates with tunable mechanical properties. Curr Protoc Cell Biol Chapter 10, Unit 10 16.
